# BCG Moreau Vaccine Safety Profile and NK Cells—Double Protection Against Disseminated BCG Infection in Retrospective Study of BCG Vaccination in 52 Polish Children with Severe Combined Immunodeficiency

**DOI:** 10.1007/s10875-019-00709-1

**Published:** 2019-11-20

**Authors:** Ewa Bernatowska, Małgorzta Skomska-Pawliszak, Beata Wolska-Kuśnierz, Małgorzata Pac, Edyta Heropolitanska-Pliszka, Barbara Pietrucha, Katarzyna Bernat-Sitarz, Nel Dąbrowska-Leonik, Nadia Bohynikova, Barbara Piątosa, Anna Lutyńska, Ewa Augustynowicz, Ewa Augustynowicz-Kopeć, Maria Korzeniewska-Koseła, Maria Krasińska, Katarzyna Krzysztopa-Grzybowska, Anna Wieteska-Klimczak, Janusz Książyk, Teresa Jackowska, Mirjam van den Burg, Jacques J. M. van Dongen, Jean-Laurent Casanova, Capucine Picard, Bożena Mikołuć

**Affiliations:** 1 Department of Immunology, The Children’s Memorial Health Institute, Warsaw, Poland; 2 Histocompatibility Laboratory, Children’s Memorial Health Institute, Warsaw, Poland; 3 Department of Medical Biology, The Cardinal Stefan Wyszyński Institute of Cardiology, Warsaw, Poland; 4 Department of Epidemiology, National Institute of Public Health – National Institute of Hygiene, Warsaw, Poland; 5 Department of Microbiology, National Tuberculosis Reference Laboratory, National Tuberculosis and Lung Diseases Research Institute, Warsaw, Poland; 6 Department of Tuberculosis Epidemiology and Surveillance, National Tuberculosis and Lung Diseases Research Institute, Warsaw, Poland; 7 Department of Tuberculosis and Lung Disease, Mazovian Centre for Tuberculosis and Lung Disease, Otwock, Poland; 8 Department of Sera and Vaccines Evaluation, National Institute of Public Health – National Institute of Hygiene, Warsaw, Poland; 9 Department of Paediatrics, Nutrition and Metabolic Diseases, Children’s Memorial Health Institute, Warsaw, Poland; 10 Department of Paediatrics, Medical Centre of Postgraduate Education, Warsaw, Poland; 11 Department of Paediatrics, Bielanski Hospital, Warsaw, Poland; 12 Department of Immunohematology and Blood Transfusion (IHB), Leiden University Medical Center (LUMC), 2333 Leiden, ZA Netherlands; 13 St. Giles Laboratory of Human Genetics of Infectious Diseases, Rockefeller Branch, The Rockefeller University, New York, USA; 14 Laboratory of Human Genetics of Infectious Diseases, Necker Branch, INSERM UMR 1163, Necker Hospital for Sick Children, Paris, France; 15 Paediatric Hematology-Immunology Unit, Necker Hospital for Sick Children, Paris, France; 16 Howard Hughes Medical Institute, New York, NY USA; 17 Paris Descartes University, Imagine Institute, Paris, France; 18 Study Centre for Primary Immunodeficiency, Necker-Enfants Malades Hospital, Assistance Publique des Hôpitaux de Paris (AP-HP), Paris, France; 19 Department of Paediatrics, Rheumatology, Immunology and Metabolic Bone Diseases, Medical University of Bialystok, Białystok, Poland

**Keywords:** Primary immunodeficiencies, BCG Moreau vaccine, disseminated BCG infection, NKcells

## Abstract

**Objectives:**

The aim of the study was to estimate the rate of adverse reactions to live BCG Moreau vaccine, manufactured by Biomed in Poland, in severe combined immunodeficiency (SCID) patients.

**Material:**

The profiles of 52 SCID patients vaccinated at birth with BCG, hospitalized in Children’s Memorial Health Institute, Warsaw (CMHI), in the years 1980–2015 were compared with those of 349 BCG-vaccinated SCID patients from other countries analyzed by Beatriz E. Marciano et al. in a retrospective study (Marciano et al. J Allergy Clin Immunol. 2014;133(4):1134–1141).

**Results:**

Significantly less disseminated BCG infections (10 out of 52 SCID, 19%) occurred in comparison with Marciano study—119 out of 349, 34% (*p* = 0.0028), with no death in patients treated with SCID anti-TB drug, except one in lethal condition. In our study, disseminated BCG infection was observed only in SCID with T-B+NK- phenotype and significantly lower NK cell counts (*p* = 0.0161). NK cells do not influence on the frequency of local BCG reaction. A significantly higher number of hematopoietic stem cells transplantations (HSCT) were performed in CMHI study (*p* = 0.0001). Anti-TB treatment with at least two medicines was provided.

**Conclusion:**

The BCG Moreau vaccine produced in Poland, with well-documented genetic characteristics, seems to be safer than other BCG substrains used in other regions of the world. Importantly, NK cells seem to play a role in protecting SCID patients against disseminated BCG complications, which NK- SCID patients are more prone to. HSCT and TB therapy could be relevant due to the patients’ survival and the fact that they protect against BCG infection.

**Electronic supplementary material:**

The online version of this article (10.1007/s10875-019-00709-1) contains supplementary material, which is available to authorized users.

## Introduction

BCG vaccination administered at birth or shortly after birth is included in vaccination schedules in countries with a high prevalence of tuberculosis (TB). At present, three different BCG vaccine substrains, Danish 1331, Tokyo 172–1, and Russian BCG-I, are recommended by the World Health Organization (WHO) as International Reference Reagents (IRR). These three substrains constitute the major proportion of BCG vaccine production worldwide as they are supplied by the United Nations Children’s Fund (UNICEF) [1–3]. An additional substrain, Moreau-RJ, was approved as a WHO IRR by the WHO Expert Committee on Biological Standardization meeting in 2012 [1, 2]. In general, the majority of locally produced BCG vaccine substrains have not been well-characterized. In terms of efficacy, no BCG substrain was found markedly superior to other strains and there is no global consensus on the choice of an optimal BCG substrain for general use [3–5].

Differences in reactogenicity between vaccines can be observed, and they relate to particular substrains. In 1955, the highly reactogenic BCG Danish vaccine was replaced in Poland by the locally produced BCG Moreau vaccine, a descendant of Brazilian BCG Moreau substrain. The BCG Danish 1331 vaccine and the Pasteur substrains have generally been considered reactogenic [6–10]. The mean risk of osteitis following BCG vaccination varies from country to country, with some reports indicating low incidence (e.g., 0.02 per million in Japan) and some presenting it as high (e.g., 1000 per million in Sweden and Finland) [5, 7–9]. In 1971, a dramatic rise in the incidence of osteitis observed in Sweden and Finland coincided with the replacement of the Gothenburg substrain of the BCG vaccine produced by the Swedish BCG Laboratory, based on the same Danish 1331 substrain [8]. In 1978, it was replaced by the BCG Glaxo substrain, which caused lower incidence of BCG osteitis in Finland and in Britain [6–8].

On the basis of the results obtained by Zapaśnik-Kobierska and Stopnicka, lymphadenitis complications in newborns vaccinated with BCG Moreau, BCG Danish 1331, or BCG Pasteur in the vaccinated cohort were found to stand at 0.3%, 2.4%, and 4.9%, respectively [11]. Furthermore, trials performed in Poland in 1972 revealed that the BCG Pasteur vaccine produced by the Lublin manufacturer was less reactogenic than that originally produced in France (18.6% versus 28.4%) [10]. Compared with the Pasteur substrains, the Tokyo and Moreau substrains are rarely associated with a high incidence of adverse events [5, 9, 10]. A recently published randomized clinical trial of a BCG vaccine produced with the Danish substrain 1331 SSI showed a higher incidence of lymphadenitis than expected, with a regional lymphadenitis rate of 6.1 per 1000 vaccinated [12]. Lymphadenitis following immunization with the BCG Moreau vaccine in Poland in the years 1994–2000 was estimated to be 0.2 per 1000 vaccinated. It was reported that the incidence rate of suppurative lymphadenitis in Poland was lower in comparison with other countries [13].

The frequency of fatal BCG disseminated infection is estimated at approximately 0.06–1.56 cases per million doses of vaccine administered, typically in primary immunodeficiency disease (PID) [14–34]. The majority of BCG disseminated infections have been reported in the literature in patients highly susceptible to mycobacterial diseases, well-defined SCID patients and those with Mendelian susceptibility to mycobacterial diseases (MSMD) [15–27]. The fatal outcome of BCG disseminated infection has also been reported in other PID less prone to BCG infection, such as chronic granulomatous diseases (CGD), hyper-IgE syndrome (HIES), X-linked hyper IGM syndrome (HIGM), nuclear factor (NF)-κB essential modifier (Nemo), and GATA2 deficiency [15, 18, 21, 22, 27–29]. One of the biggest groups of PID patients with BCG disseminated infection was retrospectively analyzed by Casanova et al. [14–16]. A multicenter, retrospective study comprising the data obtained from 28 centers in 17 different countries has recently been published by Marciano et al. This is the largest study published to date, involving 349 SCID patients vaccinated with BCG vaccine during the first months of life [35].

The aim of the present study was to estimate the frequency of complications and the risk associated with BCG vaccination in SCID patients hospitalized in the Department of Immunology, CMHI in Warsaw, over the period of 35 years, and to compare the results with those described in the retrospective study of SCID patients vaccinated with a variety of BCG vaccines containing different substrains used worldwide [35].

## Material and Methods

As many as 1727 patients were diagnosed with PID in the Department of Immunology, CMHI, Warsaw, between 1980 and 2015. A total of 52 Caucasian origin SCID patients, born in Central-Eastern Poland, were vaccinated with BCG at birth. The incidence of BCG-associated infections following vaccination with the BCG Moreau vaccine produced by Biomed, Lublin, Poland, was analyzed in a group of SCID patients susceptible to BCG infection. BCG disseminated infections were diagnosed based on clinical, microbiological, and histopathological findings following ESID diagnostic criteria [19] (https://esid.org/Working-Parties/Registry-Working-Party/Diagnosis-criteria). Localized BCG infection was referred to as a lesion inside the inoculation site (> 10 mm) and/or lymphadenitis limited to the region of the inoculation site while disseminated BCG infection was defined as a persistent process spreading over two or more regions beyond the inoculation site. The *Mycobacterium tuberculosis complex* molecular analysis was conducted using the PCR (MTD Gen-Probe) test and the analysis of *mycobacterium* culture was performed in a BACTEC 460 Tb or MGIT 960 system. Mutation analysis in PID patients was performed at the Department of Immunology, Erasmus MC in Rotterdam, Netherlands, in the Center for the Study of Primary Immunodeficiencies, Assistance Publique, Hopitaux de Paris, Necker Hospital, 75,015 Paris, France. Absolute numbers and percentages of circulating B and T subsets CD19/CD20, CD3, and natural killer cells (NK-CD56/CD16) were assessed and compared with reference values established in age-matched groups of healthy children [36]. Statistical analyses were performed using STATISTICA v 10.0 and Microsoft Excel v 2007. Quantitative variables were characterized by the arithmetic mean of standard deviation or median or max/min. Qualitative variables were presented as counts and percentages. In order to check if a quantitative variable derived from the population of normal distribution, the Shapiro-Wilk test was used. Statistical significance of differences between groups (unpaired variables model) was processed with the Student *t* test or Mann-Whitney *U* test. The Chi-squared test and Fisher’s exact test for independence was used for qualitative variables. In order to determine dependence, strength and direction between variables, correlation analysis was used by determining the Pearson or Spearman’s correlation coefficients. In all calculations, the statistical significance level of *p* = 0.05 was used. The statistical analysis of the retrospective study of 52 SCID patients hospitalized at CMHI was compared with that of 349 SCID patients investigated by Beatriz E. Marciano and co-authors [35].

### Ethics Statement

This study was approved by the Bioethics Committee of CMHI—resolution no 51/KBE/2017, 06 September 2017.

## Results

In 52 SCID patients diagnosed in CMHI, the mean age at which first SCID symptoms occurred was 3.3 months (in the range of 1.0–24.0), the age of SCID diagnosis was 13.5 months (in the range of 1.0–63.0). In 19 out of 52 cases, the diagnosis with SCID was carried out at the age of 7–12 months, which was comparable with 94 patients (*p* = 0.1510) from the Marciano study.

Disseminated BCG infection occurred in 10 SCID patients out of 52 vaccinated with BCG, twelve localized complications were present in both group, separately or together with disseminated BCG infection. (Table S1). Disseminated BCG infection was diagnosed in 10 SCID patients with TB+NK-phenotype, in 6 with IL2RG mutation, in one patient with JAK3 deficiency and in 3 with unknown mutations. Disseminated BCG infection was not present in the group of TB-NK+SCID patients, including 16 patients with RAG1/2, 2 patients with IL7RA and 2 with Artemis mutation, 1 patient each with MHC II, ZAP-70, and Cernunnos, and in 10 patients with unknown mutations (Table S1). Among SCID patients, the clinical manifestation of disseminated BCG infection was recognized as meningitis in 1 patient, bronchopneumonia in 4, disseminated skin infection in 3, single skin abscess in 2, and osteomyelitis and/or pathological fractures occurred in 6 patients. Infiltrations in spleen and liver abscess were observed in 5 patients. No brain abscess, lymphadenitis, and other localization of BCG infection were observed. The infection was confirmed with the positive polymerase chain reaction (PCR) for the *Mycobacterium tuberculosis* complex and culture in 8 out of 10 patients. Following the diagnostic criteria for disseminated BCG infection, possible diagnosis of disseminated BCG infection was established in two patients with clinical symptoms and typical histopathologic changes with granulomas formation diagnosis [19] (https://esid.org/Working-Parties/Registry-Working-Party/Diagnosis-criteria). In three infants, SCID diagnosis was established before 1985, when they were admitted in the terminal stage to the intensive care unit. Two of them died soon in the course of multiple organs insufficiency, postmortem examination found granulomas, which is the full field diagnosis criteria of possible form of disseminated BCG infection [19] (https://esid.org/Working-Parties/Registry-Working-Party/Diagnosis-criteria). The third one, with a positive family history (death of 5 infants, boys) was admitted because of respiratory insufficiency and hepatosplenomegaly. Bronchopulmonary lavage samples were tested for *Mycobacterium tuberculosis* complex; positive PCR and culture results led to the diagnosis as a definitive form of the disease [19] (https://esid.org/Working-Parties/Registry-Working-Party/Diagnosis-criteria). He died after 4 months, despite the administration of 5 anti-TB medications in a course of overwhelming BCG infection. The HSCT procedure was not considered because of serious condition of this child.

Disseminated BCG infections in 10 (19%) SCID patients hospitalized at CMHI constitute a significantly lower score in comparison with 119 (34%) in the Marciano study (*p* = 0.0028). However, in 12 (24%) SCID patients with localized BCG, the rate was higher, but not significantly, compared with the Marciano study’s 60 (17%) (*p* = 0.2417). Mortality caused by BCG-associated complications in the Marciano cohort of SCID patients was higher, but not significantly: 46 (13%) deaths out of 349 (*p* = 0.1724). In the CMHI cohort, 3 (5%) deaths occurred before 1985.

The median absolute number of T cells at the time of SCID diagnosis in patients with disseminated BCG-associated complications was found to be insignificant (*p* = 0.0915), while the absolute number of B cells was significantly higher (*p* = 0.0126) and the number of NK cells was significantly lower (*p* = 0.0161) in the group with BCG complications in the CMHI study (Table S2).

Disseminated BCG-associated complications in the CMHI study were found only in 18 out of 52 SCID patients with a low number of NK cells (*p* < 0.000001), localized complications were observed in both groups; insignificantly higher in NK+SCID patients (*p* = 0.4859) (Table S3).

Dual drug anti-TB therapy (isoniazid and rifampicin) was administered to 17 patients (33%) with local BCG complications. In 7 out of 10 SCID patients, BCG disseminated infection was successfully treated in all of them with 4 or 5 anti-TB drugs (isoniazid, rifampicin, ethambutol, ciprofloxacin, and aminoglycoside). In our study, the mean age at HSCT was 21.4 m. (a range of 4.0–96.0 m.). HSCT was administered in 43 patients (83%) in total vs 190 (54%) SCID patients in the Marciano study, the number being significantly higher in the CMHI study (*p* = 0.0001). Fifty-eight out of 349 SCID patients were diagnosed in Brazil, constitute the largest cohort of this study [35]. Similarly, fewer HSCT procedures were performed in SCID patients in Brazil, in 24 (46%) than at CMIH, in 43 (83%) (*p* = 0.0001). Polish and Brazilian cohorts were vaccinated at birth with a locally produced BCG Moreau vaccine [18, 19, 35].

In 7 SCID patients with BCG disseminated infection HSCT procedure was introduced: in 1 an unconditioned transplant from the matched related brother was done, in 4 patients HSCT from no HLA-matched related donor was performed, in 2 -HSCT from HLA-matched unrelated donor was done. In all 6 patients reduced-intensity conditioning was used, partial ablation with chemotherapy, serotherapy, antithymocyte globulin. This conditioning regimen prevented graft rejection and graft versus host disease (GVHD) with a satisfying stem cell engraftment, in one patient transient, moderate course of this disease was observed. Mixed chimerism concerning various cell lines was observed in 4 infants and is still present in one. Antimycobacterial treatment was discontinued within 8–15 months in all 7 treated patients, which coincided with immunological reconstitution. In 2 post-HSCT patients local and disseminated reactivation of BCG occurred within 3 months after transplantation. One of the patients was treated with 2 anti-TB drugs because of local BCG infection. He began to lose weight and a subfebrile condition developed. Infiltrations in spleen, liver, pathological fracture of the right hand third and fourth finger distal bones and disseminated skin abscess appeared. During the next 15 months, clinical improvement and full chimerism occurred. In the other patients with local BCG infection following HSCT, treatment with 2 anti-TB drugs was discontinued within the next few weeks. Enlargement and infiltration in the occultation site was observed and slowly dispersed during the 8 months of therapy with 3 anti-TB drugs.

## Discussion

In the last three decades, numerous studies on BCG complications in PID patients have been published by national and regional or local centers. The cumulative study of 349 patients with SCID from 17 countries vaccinated with BCG constitutes the biggest record published to date [35]. The present paper compares the clinical course of disseminated BCG infection in 52 vaccinated SCID patients hospitalized at CMHI, Warsaw, with that documented in the study of Marciano et al. [35].

### Reactogenicity of BCG Substrains

Throughout the years, a great number of publications on BCG-associated complications in PID patients susceptible to this infection have reflected the differences in the reactogenicity of various BCG vaccines produced with different substrains [14–34]. A summary of the earliest national retrospective studies performed in France between 1951 and 1994 reported 121 disseminated BCG infections in PID patients [15]. French studies conducted between 1974 and 1994 noted the deaths of 8 out of 16 SCID patients vaccinated with the BCG Pasteur substrain and 2 other patients vaccinated with the Glaxo and Copenhagen substrains, respectively [16]. A single-center French study conducted in the years 1970–1992 reported the occurrence of disseminated BCG infection in 10 out of 28 patients vaccinated with BCG in the group of 117 investigated PID patients [16]. In Canada, routine vaccination at birth with the BCG Pasteur Merieux Connaught Vaccine has been limited to some First Nation communities. In the years 1993–2001, four infants with SCID and one with HIV infection, vaccinated before the age of 3 months, developed disseminated BCG infection and subsequently died [30]. A retrospective study conducted between 2007 and 2012 in China presented the clinical outcome of PID patients vaccinated at birth with locally produced vaccines obtained from four different manufacturers using the BCG Danish substrain for production [21]. Fourteen out of 74 patients in this study, including 32 well-defined PID patients, developed disseminated BCG infection, two of SCID patients died. In the Czech Republic, 9 out of 12 SCID patients vaccinated at birth with a locally produced BCG Danish substrain vaccine developed disseminated BCG infection; five of them subsequently died [31]. Previous, as well as recent studies, demonstrate that the BCG Danish and Pasteur Merieux Connaught substrains are the most reactogenic substrains in both healthy and immunocompromised individuals [7–27]. Even in the study by Marciano et al., the Danish substrain gave the greatest, but not significant number of disseminated BCG complications [35].

The safety profile of the Polish Moreau BCG vaccine in SCID in comparison with other substrains used worldwide has been documented.

In Africa, where the BCG Danish substrain vaccine is widely used, the risk of disseminated BCG infection is several hundred-fold higher in HIV-infected infants compared to the documented risk in HIV-uninfected children [1, 37, 38]. Since 2004, WHO has not recommended BCG vaccination to neonates with HIV infection, vaccination should be delayed until anti-retroviral therapy has been initiated and they are immunologically stable [39, 40].

### Polish BCG Moreau Substrain Vaccine

The Moreau BCG substrain used in vaccine production in Poland is a descendant of the parent *Mycobacterium bovis* BCG Moreau Rio de Janeiro substrain [41–44]. A retrospective study of a large cohort of Brazilian SCID patients vaccinated at birth with the BCG Moreau/Rio de Janeiro substrain vaccine manufactured in Brazil was published by Mazzuchelli et al. [20]. The majority of those patients were included in the Marciano et al. publication [35]. Out of 60 SCID patients vaccinated with BCG, 39 presented complications related to the BCG vaccine and disseminated BCG infection was diagnosed in 29 out of 60 (48.3%) [20]. By contrast, in our study disseminated BCG infection was observed only in 10 (19%) out of 52 SCID patients vaccinated with the Polish BCG Moreau substrain vaccine*.* Since various BCG substrains have been associated with different reactogenicity, genetic variations might also be involved in the level of potential BCG complications [42–44]. Krysztopa-Grzybowska et al. evaluated the genomic stability of the BCG Moreau substrain of the seed lots used in BCG vaccine production in Poland, demonstrating the reproducibility of genetic profiles [42–44]. The authors found that a defect in the biosynthesis of PDIM/PGL in the BCG Moreau substrain might reflect the lower level of reactogenicity of this vaccine. Additionally, unlike the Rio de Janeiro parent BCG substrain, the BCG Moreau substrain used in Poland does not harbor a deletion in *Rv3887c*, a region involved in the membrane transport protein which constitutes part of the ESX-2 type VII secretion system. The role of *Rv3887c* is not well known, but homology with the components of the ESX-1 secretion system might influence immunogenicity [45]. These findings suggest that the distribution of the BCG Moreau substrains for their subsequent use in vaccine production in different parts of the world might influence the divergent microevolution of the BCG Moreau substrain used in Brazil and Poland [42–45]. The documented genetic characteristics of the BCG Moreau vaccine produced in Poland and its good safety profile, as compared to other BCG vaccine substrains, are important attributes which should warrant its inclusion in the Expanded Programme on Immunization (EPI). Different BCG vaccine substrains have been obtained as a result of genetic changes which occurred during repeated subculture in different countries. Genetically divergent BCG vaccine substrains might be associated with different protective efficacy against tuberculosis, different rates of adverse events and the necessity of anti-TB treatment.

### Therapy in SCID

Immunological reconstitution by HSCT and treatment of mycobacterial infection are the key elements of successful therapy in SCID patients. HSCT as the major type of intervention affecting the survival rates in patients with SCID was also confirmed by Marciano et al. [35]. In the SCID patients investigated in our study, significantly fewer cases of disseminated BCG infection were observed in comparison with the Marciano et al. study, which could be related to a significantly low number of HSCT procedures implemented in SCID patients in the latter [35]. The authors observed that considerably fewer HSCT procedures were implemented in the Brazilian cohort of the study. A separate study of 70 Brazilian SCID patients confirmed that fewer than half of the patients (42%) received HSCT; the mortality rate was as high as 35.5%, caused by BCG disseminated infection in at least 29% of cases [20]. BCG complications are the serious health problem in all SCID worldwide and are an indication for immediate anti-TB treatment and implementation of HSCT. Early diagnosis of the symptoms is crucial; without diagnosis and implementation of relevant treatment, the disseminated BCG disease may be the cause of death. BCG complication following HSCT is relatively rare. In all 7 patients with BCG disseminated infection, HSCT together with anti- TB therapy was attempted as the only available curative treatment, administered regardless of the occurrence of: related, unrelated donors, conditioned or unconditioned transplant, full chimerism or mixed chimerism. The practical recommendation resulting from our observations is not to discontinue anti-tuberculosis treatment in the post-transplant period, despite the increasing parameters of cellular immunity. Discontinuation of anti-TB treatment may take place when the clinical symptoms of BCG infection subside. What is critical in the treatment of both local and disseminated complications of BCG infection is the early implementation of at least two anti-TB drugs administered as soon as local BCG complications appear [19] (https://esid.org/Working-Parties/Registry-Working-Party/Diagnosis-criteria). Anti-TB therapy is continued after HSCT, when local or disseminated symptoms are present or reactivation of BCG infection occurred, usually a few weeks after transplantation. Isoniazid and rifampicin constitute a base treatment in local complication, if disseminated process occurred after other anti –TB drugs were administered. In our group of SCID with BCG – associated complication, treated with anti-TB drugs, full recovery was observed, with no relapse during long-lasting observation, except one patient from early 80s in whom anti-TB treatment was ineffective, because of the severity of the overwhelming BCG infection.

### Immune Defense Against *Mycobacterium bovis* BCG Infection

The immune response to *Mycobacterium tuberculosis* infection involves a complex cellular process which is not fully understood. Protection from disseminated disease in children is associated with a Th1-type response involving CD4+ T cells which produce interferon-gamma (IFN-γ). The study by Marciano et al. revealed that a lower number of T cells in SCID patients increased the probability of them developing BCG-associated complications. In our study, the number of T cells did not have an impact on the occurrence of disseminated BCG in any type of patient diagnosed with SCID (Table S1,2). The number of B cells in SCID does not play a role in the defense against pathogens, since B cells, if present, do not produce antibodies [17].

NK cells can provide the primary source of IFN-γ production during blood exposure to the *Mycobacterium bovis* BCG substrain and thus contribute to the non-specific beneficial effects of BCG vaccination [46]. The production of IFN-γ by NK cells following direct contact with *Mycobacterium bovis* BCG in the absence of accessory cells has also been observed by other investigators [47]. The IL-12/IFN-gamma axis is crucial for protective immunity against Mycobacterium in humans and mice. The relative contribution of various human blood cell subsets and molecules to the production of or response to IL-12 and IFN-gamma showed that monocytes were probably the main producers of IL-12, and that NK and T cells produced similar amounts of IFN-gamma [47]. IFN-γ production by CD56 cells reflects the major function of NK cells, also presenting the immunotherapeutic benefits observed for *Mycobacterium bovis* BCG against malignancies [48]. It has also been demonstrated that BCG revaccination of adults with latent *Mycobacterium tuberculosis* infections induces long-lived BCG-reactive NK Cell responses [49]. The investigation of our 18 patients with SCID *RAG1* and *RAG,* and TB-NK+ revealed that the presence of NK cells may provide a defense against the development of disseminated BCG infection, which confirms the important role of NK cells in the defense against *Mycobacterium bovis* BCG infection. However, Marciano study shows that patients with and without BCG- associated complications were not significantly different in natural killer cell number [35].

Our findings demonstrate that the non-specific immune response evoked by NK cells protects against the overwhelming mycobacterial infection not only in RAG1/2 SCID deficiency patients, but also other SCIDs with the presence of NK cells (Table S1). In a multicenter study, in SCID patients with a recombination-activating gene RAG1/2 mutation, vaccinated at birth with BCG vaccines containing a variety of *Mycobacterium bovis* substrains, only local BCG complications appeared. In all but one patient, the absolute number of NK cells was within the normal range of 0.2–1.2 × 10E9/L [50]. However, BCG disseminated infection can rarely occur in TB-NK+ SCID patients with RAG1/2 mutation and patients vaccinated with the BCG Moscow-368 vaccine and the BCG Sofia SL 222 vaccine manufactured in Bulgaria, derived from the Moscow-368 substrain and used in Ukraine since 2014 [51]. The important role of innate immune response to mycobacterial infection has also been demonstrated in a group of newly described PID, particularly those with gain-of-function STAT1 mutation, where decreased STAT1-mediated IFN -γ responses impair immunity against mycobacterial infection [34, 52].

In our study, disseminated BCG infection was observed only in SCID patients with the IL2RG and JAK3 deficiency (T-B+NK-) (Table S1). Defects impairing cellular immune response, phagocytic function, and IFN-γ production are associated with BCG complications. Our findings demonstrate the major role played by the number of NK cells in preventing BCG complications as compared to the number of T cells.

In summary, for more than 50 years of the BCG Moreau vaccine being produced in Poland, with its confirmed genetic changes, it has proven to possess a well-documented safety profile, recently also confirmed in our paper, in comparison with the Rio de Janeiro parent BCG substrain and to the Danish and Pasteur substrains [41–44, 49, 50]. It was confirmed by significantly fewer number of complications with disseminated BCG in our SCID patients and in other less susceptible patients in comparison with different BCG vaccines substrains used worldwide [18, 19, 28, 33, 49, 50]. Our findings show that lower T cell numbers do not have a decisive effect on the spread of disseminated BCG infection, in contrast to the findings of Marciano et al., who demonstrated significantly low T cell numbers to be predictors of such a serious infection [35]. Furthermore, statistically higher NK cell counts in our study group were revealed to provide essential protection against disseminated BCG infection to all SCID patients.

## Electronic supplementary material

ESM 1 (DOCX 26 kb)
